# Improved Exciton Dissociation at Semiconducting Polymer:ZnO Donor:Acceptor Interfaces via Nitrogen Doping of ZnO

**DOI:** 10.1002/adfm.201303994

**Published:** 2014-03-07

**Authors:** Kevin P Musselman, Sebastian Albert-Seifried, Robert L Z Hoye, Aditya Sadhanala, David Muñoz-Rojas, Judith L MacManus-Driscoll, Richard H Friend

**Affiliations:** Department of Physics University of Cambridge Cavendish LaboratoryJJ Thomson Ave Cambridge, CB3 0HE, UK E-mail: kpdm2@cam.ac.uk; Department of Materials Science & Metallurgy University of Cambridge27 Charles Babbage Road, Cambridge, CB3 0FS, UK; Instituto de Ciencia de Materiales de Barcelona ICMAB-CSIC, Campus de la UABBellaterra, 08193, Spain

**Keywords:** hybrid photovoltaics, P3HT, zinc oxide, doping, Kelvin probe microscopy

## Abstract

Exciton dissociation at the zinc oxide/poly(3-hexylthiophene) (ZnO/P3HT) interface as a function of nitrogen doping of the zinc oxide, which decreases the electron concentration from approximately 10^19^ cm^−3^ to 10^17^ cm^−3^, is reported. Exciton dissociation and device photocurrent are strongly improved with nitrogen doping. This improved dissociation of excitons in the conjugated polymer is found to result from enhanced light-induced de-trapping of electrons from the surface of the nitrogen-doped ZnO. The ability to improve the surface properties of ZnO by introducing a simple nitrogen dopant has general applicability.

## 1. Introduction

Metal oxide/conjugated polymer interfaces have proven to be of great importance for a variety of devices including bulk-heterojunction solar cells,[1,2] hybrid solar cells,[3–6] light-emitting diodes,[7] and transistors.[8] Surprisingly, despite their extensive use, questions remain in regards to the photophysical processes at these interfaces. Zinc oxide/poly(3-hexylthiophene) interfaces (ZnO/P3HT), for example, have been widely used in bulk-heterojunction[2] and hybrid solar cells,[3,5,6] but the ability of this type-II junction to act as a exciton-dissociating interface has been the subject of contrasting reports. Photo-induced absorption (PIA) measurements of solution-cast ZnO/P3HT devices, for example, indicated that when functionalized P3HT was used to ensure intimate mixing of the two phases, all singlet excitons created in the polymer were quenched by ZnO via electron transfer from the P3HT to ZnO (quantitative charge gene­ration).[3] Recently, we demonstrated internal quantum efficiencies of over 50% in ordered P3HT-ZnO structures, which indicated efficient charge generation and separation.[6] These results are consistent with modeling of P3HT/ZnO interfaces, which suggested that strong coupling between the P3HT molecule and ZnO conduction band states can occur.[9,10] In contrast, bilayer ZnO/P3HT devices typically produce significantly lower photocurrents than are achieved with all-organic bilayer devices that use a fullerene acceptor, suggesting that carrier separation at the ZnO/P3HT interface is not efficient.[11] Transient absorption measurements have shown similar polaron optical absorption (due to charges in P3HT) for ZnO/P3HT bilayers as for P3HT on glass, suggesting that ZnO is not effective at charge photogeneration with the adjacent P3HT.[12] This is consistent with microwave conductivity measurements of ZnO/P3HT, which indicated that free carrier generation predominantly occurs in the bulk of the P3HT, and that photocurrent results from subsequent injection of free electrons into the ZnO.[13] Perhaps Hsu et al. best summarized the situation when they reported in their review article that electron transfer (and subsequent recombination) at organic/oxide interfaces is “currently not well understood”.[11]

**Figure 1:**
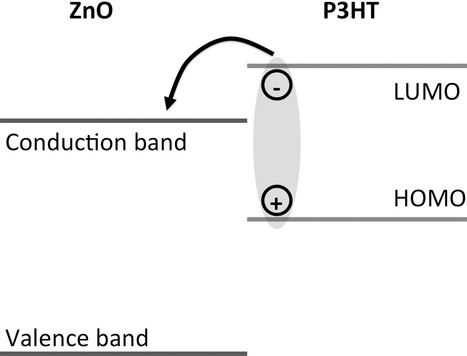
Typical energy-level diagram for the ZnO/P3HT interface, invoking bulk properties of the materials.

The contrasting nature of these reports, however, is not surprising, given the large variability in the reported properties of metal oxides. Due to its rich defect chemistry, the electrical properties of zinc oxide have been reported to range from insulating to metallic, and can be tuned by incorporating dopants.[14,15] Furthermore, the properties of the zinc oxide surface can differ greatly from its bulk properties due to the presence of surface-induced defect states within the band-gap, the adsorption of gaseous molecules, and trapping of electrons on the surface.[16–18] While bulk properties of the materials are traditionally used to describe the ZnO/P3HT interface (**Figure** **1**), it is known that the oxide's surface properties may also influence the relevant photophysical processes.[19,20] In this work, we doped zinc oxide with nitrogen (ZnO:N) to tune its electron concentration, reducing it by approximately two orders of magnitude. We studied the effect that the bulk electron concentration has on the surface properties of zinc oxide and how the surface properties influence exciton dissociation at model bilayer zinc oxide/P3HT interfaces.

## 2. Results and Discussion

### 2.1. Influence of ZnO:N on the Device Performance: a Surface or Bulk Effect?

**Figure 2:**
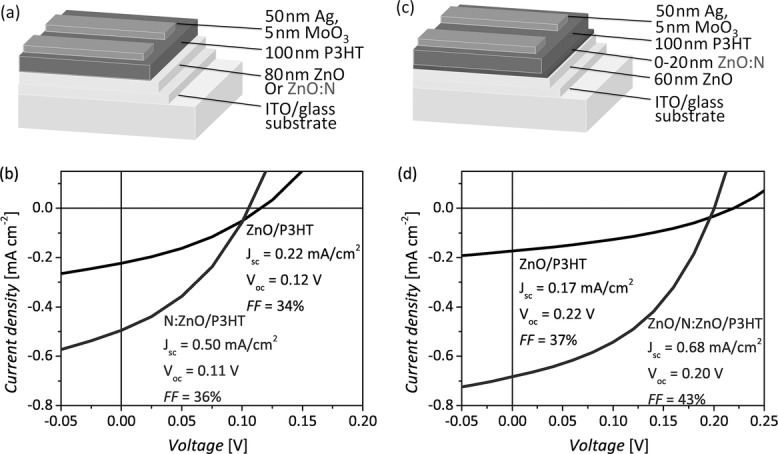
a) Schematic diagram and b) photovoltaic measurement of hybrid devices employing approximately 80 nm ZnO and ZnO:N films. c) Schematic diagram and d) photovoltaic measurement of hybrid devices employing approximately 60 nm ZnO films with and without a ZnO:N coating approximately 20 nm thick.

Bilayer solar cells were synthesized using both ZnO and ZnO:N films approximately 80 nm thick. The films were deposited using a spatial (or atmospheric) atomic layer deposition technique (AALD), where the substrate is passed underneath alternating flows of metal and oxidant precursors.[2,21,22] The cells are illustrated schematically in **Figure** **2**a and representative current ­density–voltage (*J*–*V*) curves are shown in Figure 2b. We observed a significant improvement in performance for the ZnO:N devices. In particular, the short-circuit current density (*J*_SC_) doubled with the use of ZnO:N, whereas the open-circuit voltage (*V*_OC_) decreased slightly and the fill factor (FF) improved slightly, resulting in a doubling of the power conversion efficiency.

Nitrogen is expected to introduce a shallow acceptor level (via anionic substitution) that reduces the electron concentration of ZnO, which is naturally n-type.[23,24] We have previously reported the properties of ZnO:N films deposited using this AALD technique.[25] Briefly, the films produced are polycrystalline ZnO of the wurtzite phase and the main effect of the nitrogen doping is a reduction in the carrier concentration from approximately 10^19^ cm^−3^ for the undoped ZnO to approximately 10^17^ cm^−3^ for the ZnO:N. A change in the film orientation is also observed with doping: X-ray diffraction measurements indicate a predominant a-axis orientation for the ZnO:N films, whereas the undoped films display both a-axis and c-axis contributions.[25]

The offset between the conduction band edge of the zinc oxide (measured previously to be −3.6 to −3.7 eV for ZnO and ZnO:N)[25] and the LUMO (lowest unoccupied molecular orbital) level of P3HT (typically reported to be approximately –3 eV or higher)[26] should be relatively large (>0.5 eV), such that a suitable density of unoccupied acceptor states should be available in the bulk of the ZnO for the range of carrier concentrations studied here. In fact, calculations approximating the occupancy of the ZnO conduction band states (Section S1 of the Supporting Information) indicate that for the doping range considered, electrons will only occupy a significant fraction of states within 0.05 eV of the ZnO conduction band minimum. Furthermore, the electron mobility in the ZnO:N is lower than in the ZnO,[25] such that the improvement in device efficiency is also unlikely to be due to better transport through the ZnO:N film.

Other bulk oxide properties have been proposed to explain varying charge transfer rates in hybrid systems. Tiwana et al., for example, examined dye-sensitized solar cells and found that electron injection from a Z907 dye was significantly less efficient into ZnO than into TiO_2_, and attributed this to a number of possible factors including the effective mass of electrons in the oxide conduction band (and hence the conduction band density of states), the overlap of the dye's excited-state wave function with the electron-accepting orbitals of the oxide, and the dielectric constant of the oxide.[27] Previous photoelectron spectroscopy measurements of these ZnO:N films indicated modest nitrogen incorporation (approximately 0.22 atomic percent) and negligible changes in the conduction and valence band positions,[25] such that a significant change in the effective mass of electrons in the conduction band is not expected for the ZnO:N compared to the undoped ZnO. Similarly, we do not expect the electron-accepting Zn^2+^ 4s orbitals or the dielectric constant of ZnO to be altered significantly by the incorporation of the small amount of nitrogen.

Polymer morphology is also known to be an important factor in determining interfacial charge separation and influences the optical absorption spectrum of a P3HT film.[28,29] Absorbance measurements of P3HT films on ZnO and ZnO:N substrates displayed identical spectra (Section S2 of the Supporting Information), suggesting that the difference in device performance does not follow from a different polymer morphology on the two oxide surfaces.

To investigate whether the observed photocurrent enhancement is a surface effect, rather than a bulk phenomenon, similar devices were fabricated consisting of approximately 60 nm thick ZnO layers with and without ZnO:N surface coatings approximately 20 nm thick, as shown in Figure 2c. The performance of these devices is seen to improve dramatically when the 20 nm ZnO:N coating is placed on the native ZnO surface (Figure 2d). As was observed for Figure 2b, the *J*_SC_ improved significantly with the inclusion of ZnO:N, whereas the FF and *V*_OC_ remained relatively unchanged. The data presented in Figure 2 suggests that the improved current is not attributable to the introduction of an ITO/ZnO:N or ZnO/ZnO:N interface. No ZnO/ZnO:N interface is present for the devices shown in Figure 2a,b and no ITO/ZnO:N interface is present for the devices shown in Figure 2c,d, yet both architectures exhibit improved *J*_SC_s with the inclusion of ZnO:N. This indicates that the improved charge collection observed with nitrogen doping is attributable to the ZnO:N surface and its interface with P3HT.

Furthermore, it was found that the observed *J*_SC_ improvement for the ZnO:N/P3HT interface is not specific to the particular device fabrication conditions used. Similar ZnO/P3HT and ZnO/ZnO:N/P3HT devices were synthesized using the alternative recipe described in Section 2.2, where a different P3HT was dissolved in chlorobenzene (30 mg mL^−1^) rather than o-xylene (15 mg mL^−1^), and spin-cast and annealed in air rather than in a nitrogen glovebox. *J*–*V* measurements for these devices are included in Section S3 of the Supporting Information. The current densities were lower for the cells fabricated using this recipe and the *V*_OC_s were higher, which may be attributable in part to the thicker P3HT films produced (approximately 300 nm), but a significant improvement in *J*_SC_ was again observed with the inclusion of the ZnO:N surface coating, while the other device parameters remained relatively unchanged.

### 2.2. Influence of Nitrogen Doping on the Zinc Oxide Surface

**Figure 3:**
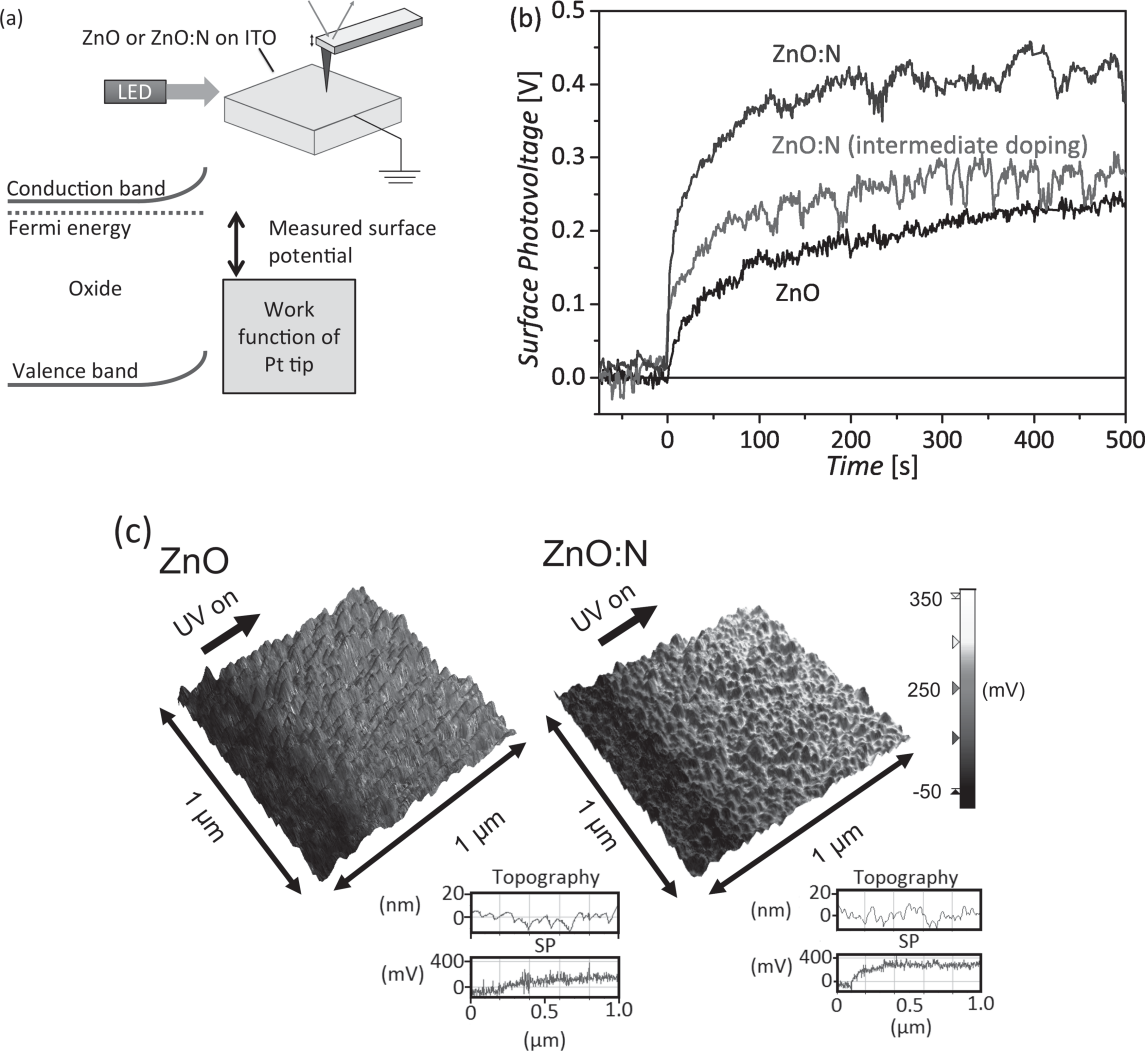
a) Schematic of the time-resolved microscopic surface photovoltage measurement and the corresponding energy level diagram. Greater trapping of electrons on the surface of the oxide is expected to increase the work function of the oxide surface and decrease the measured surface potential. b) Change in surface potential measured for ZnO, ZnO:N, and ZnO:N (intermediate doping with 10% NH_3_) upon UV illumination. c) Surface potential measurements of ZnO and ZnO:N samples superimposed on the corresponding surface topography measurement, with the position indicated where UV illumination was turned on.

To investigate how the nitrogen dopant influences the electronic surface properties of illuminated zinc oxide, a time-resolved microscopic surface photovoltage technique was employed (**Figure** **3**a). For ZnO and ZnO:N samples on ITO, the surface potential relative to a Pt-coated scanning probe tip was measured by Kelvin probe force microscopy (KPFM). The surface potential is related to the work function difference between the tip and sample, such that a larger measured surface potential is expected to correspond to a higher Fermi energy (lower work function) at the sample surface (Figure 3a). Correlating surface potential measurements to absolute work function values is complicated by the influence of adsorbed species and tip effects, such that differential measurements can be more reliably compared. We monitored the change in surface potential resulting from illumination (surface photovoltage) in a manner similar to that reported previously for other photovoltaic[30,31] and photocatalytic[32] materials.

**Figure 4:**
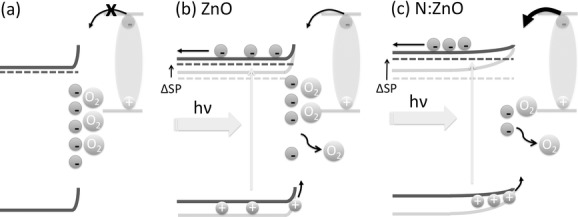
a) The trapping of electrons at the surface of ZnO results in the formation of a space-charge region. b) Photogenerated holes in the ZnO can recombine with trapped electrons, releasing adsorbed species and reducing the space charge and associated band bending at the surface. c) A thicker space-charge region is expected for the ZnO:N, which should enhance the de-trapping process and result in a greater reduction in the surface's work function upon UV illumination (as indicated by the change in surface potential, DSP).

Figure 3b shows the measured change in surface potential for approximately 80 nm thick oxide films as a function of time under UV illumination. Each data point corresponds to the average surface potential for one line scan. For the undoped ZnO, the surface potential increased gradually by approximately 200 mV over the course of several minutes. We attribute the increase in surface potential to the de-trapping of electrons from surface states and hence a decrease in the work function at the surface of the ZnO. It is well-established that the surface and grain boundaries of ZnO are prone to electron trapping and oxygen chemisorption, which result in the formation of a space-charge region, as illustrated in **Figure** **4**a.[16–18,33] The origin of surface-localized states (trapped electrons and adsorbed molecules) in ZnO is still a matter of debate,[17] and dangling bonds, steps and kinks in the lattice, as well as impurity atoms that adsorb or segregate to the surface may all contribute.[34] Under illumination, it has been reported that photogenerated holes created in the zinc oxide near its surface can recombine with trapped surface electrons, potentially releasing chemisorbed oxygen molecules, as shown in Figure 4b.[18,33,35] Notably, unlike previous reports where the change in surface potential was attributed to a large population of photogenerated electrons and observed to recover immediately when the illumination was turned off,[32] the surface photovoltage in our samples was found to decay over several hours (Section S4 of the Supporting Information). This is consistent with the persistent photoconductive behavior of ZnO, which is attributed to a slow re-trapping process following the de-trapping of electrons at grain boundaries and surfaces.[23,33,36]

For the ZnO:N sample in Figure 3b, the surface potential increased extremely rapidly, by more than 200 mV within 10 s and more than 400 mV overall. A sample with an intermediate doping (10% NH_3_ precursor) showed a similarly fast increase in surface potential when illuminated but saturated at a lower value than the ZnO:N with the highest doping level (30% NH3 precursor). KPFM scans for the ZnO and ZnO:N films are shown in the left and right images of Figure 3c respectively. The topographic measurements indicate a similar surface morphology (grain size and film roughness) for the ZnO and ZnO:N samples. The surface potential values are superimposed on the topographic data in Figure 3c and the scanning position at which the UV illumination was turned on is indicated. These measurements indicate that nitrogen doping enhances the photo-induced de-trapping of electrons, resulting in a larger reduction in the work function measured on the surface of the oxide.

The enhanced de-trapping process on the ZnO:N surface could follow from a greater concentration of photogenerated holes in the ZnO:N or more efficient transport of these holes to the surface.[34] Photothermal deflection spectroscopy measurements were performed on the ZnO and ZnO:N films (Section S5 of the Supporting Information) and indicated a larger amount of sub-band-gap absorption in the ZnO:N, which is expected to result from additional intra-gap states introduced by the dopants. However, the increase in sub-band-gap absorption was observed for ZnO:N versus ZnO, regardless of whether or not the films were annealed after deposition (as detailed in the Experimental section). Yet the non-annealed ZnO:N films did not display as large an enhancement in surface photovoltage response nor an improvement in *J*_SC_ when used in solar cells (in fact, the *J*_SC_ was reduced in this case). This suggests that greater absorption in the ZnO:N is not responsible for the enhanced de-trapping of electrons from the surface. Annealing of the ZnO:N, on the other hand, is required to enable the enhancement in de-trapping of electrons from the surface. Annealing likely removes precursor impurities and may improve dopant incorporation via diffusion from adsorbed species at grain boundaries.

The field within the space-charge region is expected to aid the de-trapping process, directing holes towards the surface and electrons away from it.[33,34] The thickness of the space-charge region should increase as the carrier concentration of the zinc oxide is reduced, such that a thicker space-charge region is expected to be formed for the ZnO:N than for the ZnO, as shown in Figure 4c. Trap state densities on the order of 10^12^ cm^−2^ have been measured for solution-processed ZnO interfaces.[37] If a similar density of electrons are trapped on the surface of our films, then for a carrier concentration of approximately 5 × 10^17^ cm^−3^ (the case for the ZnO:N films of this study), a space-charge region approximately 20 nm thick is expected to be formed, whereas for a carrier concentration of 1 × 10^19^ cm^−3^ (the case for the ZnO films of this study), a space-charge region only 1 nm thick would result. We expect that this thicker space-charge region at the surface of the ZnO:N will result in more efficient hole transport to the oxide surface and hence more efficient de-trapping of surface species (electrons and adsorbed molecules) that inhibit the transfer of photogenerated electrons from the polymer to the oxide (Figure 4c).

The equilibrium surface potential under UV illumination was found to be larger for the ZnO:N films than for the undoped films (a version of Figure 3b in which the pre-illumination surface potential values have not been normalized to zero is shown in Section S4 of the Supporting Information). Although caution must be exercised when comparing absolute values of measured surface potentials, our experiments were performed under identical conditions with the same Pt tip to enable some comparison. The larger equilibrium surface potential for the ZnO:N under illumination indicates a smaller work function for the ZnO:N surface than the ZnO surface. This is opposite of what has been observed in bulk measurements (nitrogen introduces an acceptor level that increases the work function),[25] and suggests that a lower density of trapped electrons and a smaller amount of band bending remain at the surface of the ZnO:N under illumination, consistent with what we have proposed above.

The microscopic surface photovoltage measurements were also performed with illumination from a white light-emitting diode. Despite the relatively low intensity of the illumination source and reduced absorption by the oxide films (as compared to UV illumination), a change in surface potential was observed for the ZnO:N film as before, whereas no change was seen for the ZnO (Section S4 of the Supporting Information), indicating that the de-trapping process under white light was also more efficient on the ZnO:N surface.

Finally, it is also recognized that other properties of the ZnO:N films may influence the observed de-trapping process. The nitrogen dopant may increase carrier lifetimes in the zinc oxide. In TiO_2_, for example, nitrogen doping was reported to reduce the recombination rate of electrons and holes.[32] A more mobile hole in ZnO:N would be more likely to reach the surface and de-trap an electron. Similarly, it was noted in Section 2.1 that ZnO:N exhibits a more pronounced a-axis orientation. It cannot be discounted that this orientation may be more amenable to photo-induced de-trapping of surface-localized species.

Summarizing the microscopic surface photovoltage measurements detailed above, we have observed that nitrogen doping of ZnO results in a significant change to its surface properties, particularly an enhancement in the de-trapping of electrons from the surface under illumination. We have proposed that this effect may be due to a thicker space-charge-region at the surface of the ZnO:N, different charge carrier lifetimes induced by the nitrogen dopant, different ZnO and ZnO:N film orientations, or a combination of these factors.

### 2.3. Correlating Improved Device Performance to Oxide Surface Properties

**Figure 5:**
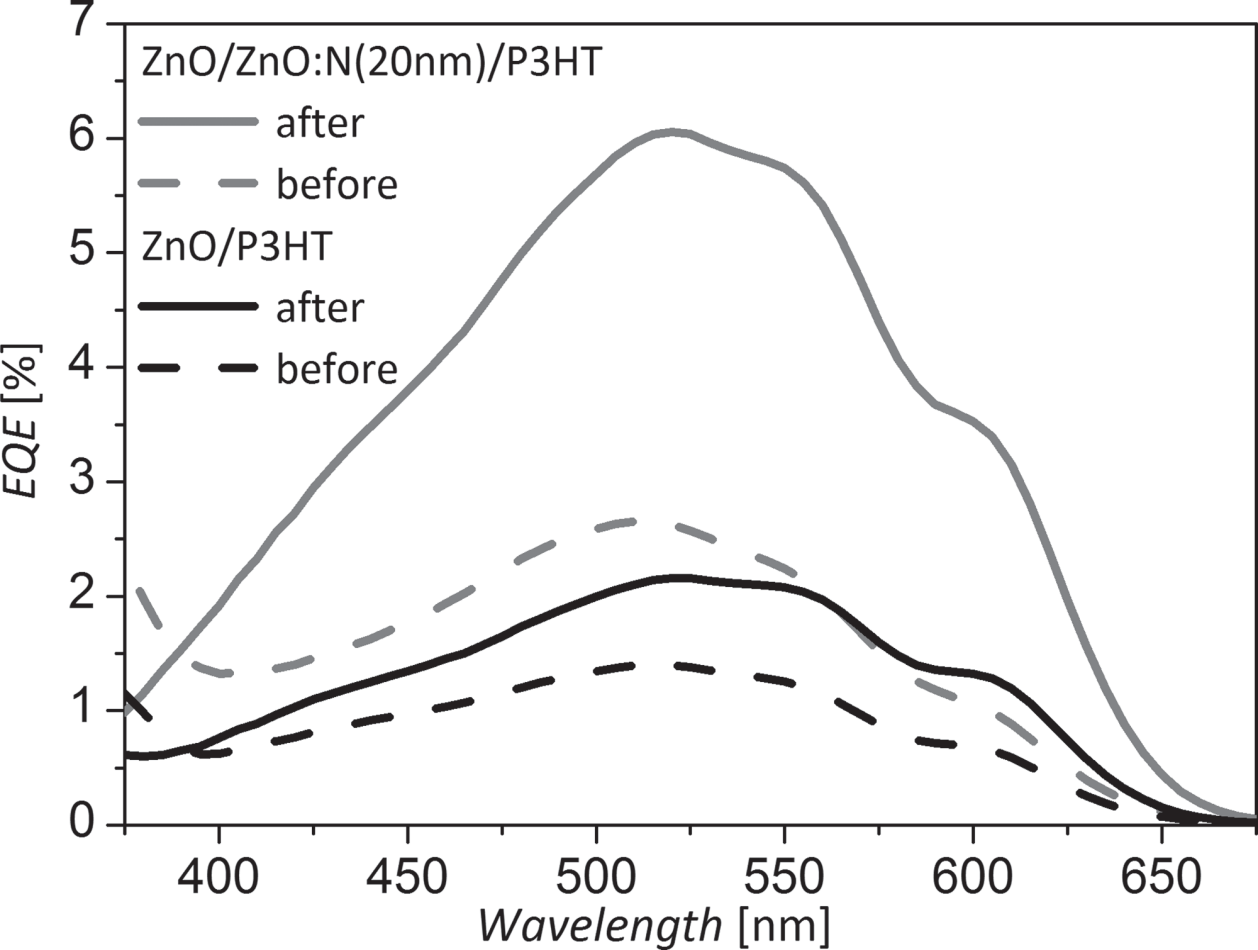
EQE measurements of ZnO/P3HT and ZnO/ZnO:N/P3HT devices after storing in the dark and after illuminating with simulated solar light.

We propose that the surface of the oxide, which is directly adjacent to the P3HT, critically influences charge transfer from the P3HT, and that the lower photocurrents obtained with the undoped ZnO surfaces in Figure 2follow from a larger density of adsorbed molecules and/or trapped electrons on the surface, which inhibit exciton dissociation and charge transfer from the P3HT to the oxide. If the improved solar cell performance we have measured for the cells incorporating ZnO:N films (Figure 2) is in fact due to more efficient photo-induced removal of surface-localized species from the ZnO:N (and subsequently more efficient exciton dissociation and charge transfer from the adjacent P3HT), we would expect to see a change in the photovoltaic performance of the nitrogen-doped devices after they have been exposed to illumination. **Figure** **5** shows the EQE of devices similar to those shown in Figure 2c (approximately 60 nm of ZnO with and without a ZnO:N surface coating) before and after illumination under simulated solar light. For the device with no surface coating, the EQE increases slightly with solar illumination, whereas for the device with the 20 nm ZnO:N coating, the EQE more than doubles *after* illumination, clearly demonstrating that the improved performance of the nitrogen-doped devices is induced by light exposure. In particular, absorption of ultraviolet light by the ZnO:N was found to be responsible for the improved performance. When a 440 nm long-pass filter was used to remove ultraviolet light from the simulated solar spectrum, the device with ZnO:N did not demonstrate an improvement in EQE after illumination for 5 min. When the filter was removed, the observed improvement in EQE was observed to take place within 1 min.

### 2.4. Examining Exciton Dissociation at the Hybrid Interfaces

We have shown that trapped electrons are more effectively removed from the surface of nitrogen-doped ZnO under illumination, which results in an enhanced photocurrent in ZnO:N/P3HT devices. However, the question remains as to whether the improved charge collection follows from more efficient dissociation of excitons formed in the P3HT, or whether the charge yield is unchanged and ZnO:N is only more efficient at collecting electrons generated within the P3HT.

**Figure 6:**
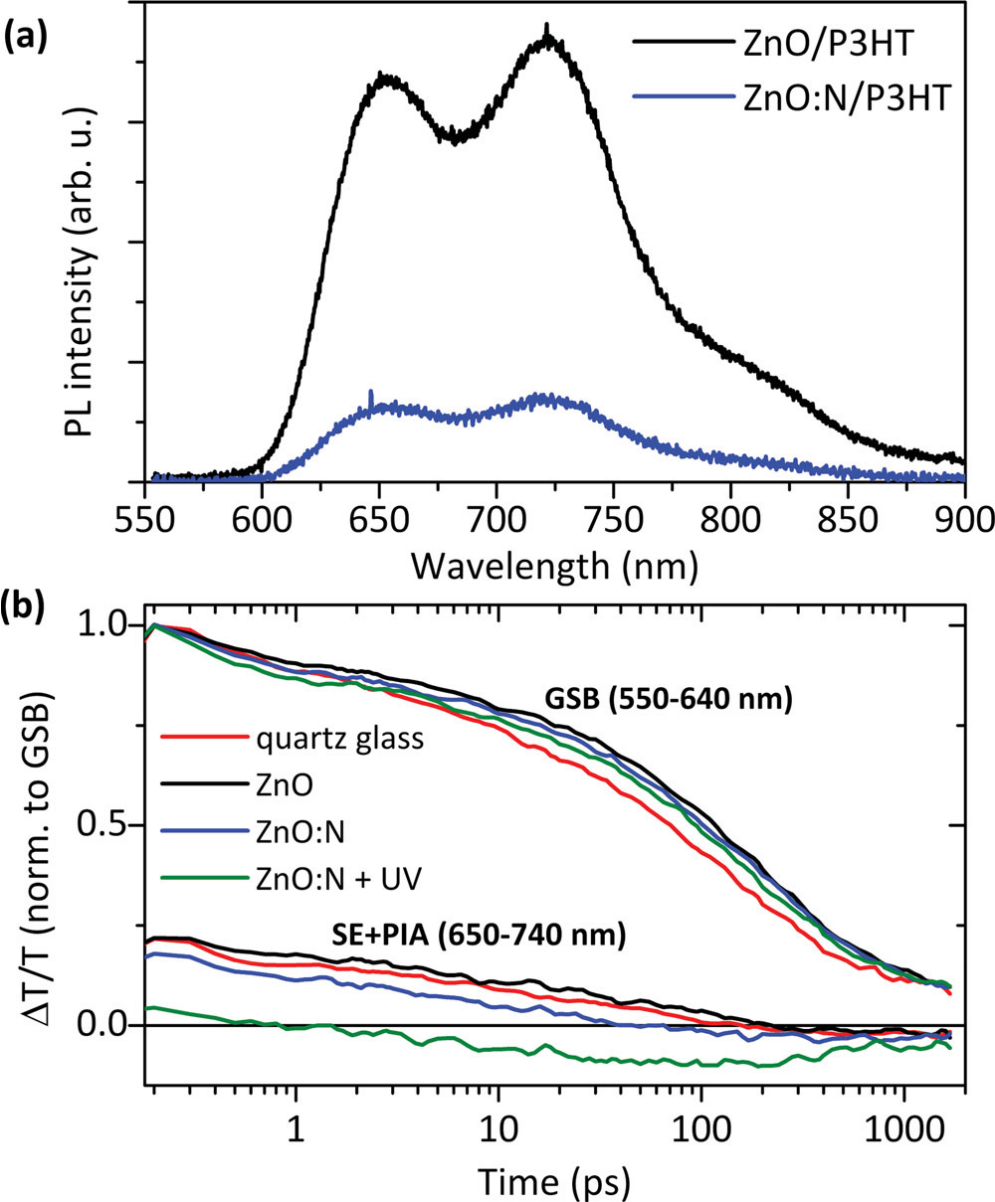
a) Photoluminescence spectra of thin (approximately 10 nm) P3HT films on ZnO and ZnO:N substrates. More significant quenching is seen for the ZnO:N substrate. b) Transient absorption kinetics for 50 nm P3HT films on ZnO, ZnO:N, and Spectrosil B quartz glass surfaces. The samples were excited at 1.5 μJ cm^−2^ at 530 nm. To compensate for slight changes in excitation intensity, the kinetics of each sample were normalized to the GSB signal intensity.

Photoluminescence quenching experiments are often used to evaluate an oxide's ability to dissociate excitons in an adjacent polymer.[11,38] Steady-state PL measurements were performed on thin (approximately 10 nm) P3HT films on ZnO and ZnO:N substrates and the spectra are shown in **Figure** **6**a. A reduction in the photoluminescence intensity is observed for P3HT on ZnO:N versus P3HT on ZnO, consistent with greater exciton quenching for the P3HT on ZnO:N. It is noted, however, that in addition to providing a quenching surface, a substrate may also influence the properties of the polymer and its non-radiative decay channels,[39] such that caution should be taken when correlating photoluminescence quenching to exciton dissociation via charge transfer from the P3HT to the oxide.

In an attempt to observe charge populations in these systems more directly, transient absorption (TA) measurements were performed on approximately 50 nm thick P3HT films on ZnO and ZnO:N coated quartz glass (Spectrosil B) substrates. This differential absorption spectroscopy technique uses an ultrashort excitation pulse to create a population of photo-excited states and a second broadband probe pulse to observe the changes in transmission (Δ*T*) through the sample as a function of the wavelength as well as the delay between the excitation and probe pulse. Different photo-excited species can be identified in the spectra and their lifetimes observed based on their differential absorption signatures. The TA spectra for the hybrid samples, as well as that of a neat P3HT film on quartz glass, are included in Section S6 of the Supporting Information.

The TA spectra of the singlet excitons in P3HT on quartz glass show two distinct spectral features: a strong bleach of the ground state absorption (GSB) from 520 to 650 nm and a stimulated emission (SE) signal at 670 to 775 nm. In contrast to the two positive features of singlet excitons, charged states in P3HT show a GSB signal up to ∼640 nm, followed by a negative photoinduced absorption (PIA) feature from 650 nm.[40] Therefore, the spectral region from 650 nm onwards allows distinguishing between the positive SE signal of singlet excitons and the negative PIA of charges, and thus characterizes the efficiency and dynamics of charge formation on different substrates.

Figure 6b shows the kinetics of the GSB (550–640 nm) and the SE/PIA region (650–740 nm) for the 50 nm thick P3HT films on ZnO, ZnO:N, and quartz glass surfaces, as well as the P3HT on ZnO:N after UV exposure. For better comparison all kinetics were normalized to the GSB signal. The kinetics in the SE/PIA region (650–740 nm) show the strongest positive signal for the neat P3HT on quartz glass and for P3HT on ZnO, indicating the population of singlet excitons is largest for these samples. The similarity in the kinetics for P3HT on quartz glass and on ZnO over the entire measured timescale suggests that the undoped ZnO does not effectively promote exciton dissociation. Examining the SE/PIA kinetics of P3HT on ZnO:N on the other hand, we see that the initial signal is significantly lower, because of a stronger negative contribution from charged states (PIA) which competes with the positive stimulated emission. An even stronger reduction in the initial signal is observed after exposing the ZnO:N sample to UV light for approximately 3 minutes under nitrogen atmosphere. In contrast, the spectra and kinetics of the neat P3HT were not affected by UV exposure. These measurements suggest that the enhanced photocurrent observed in the ZnO:N/P3HT devices is not simply due to ZnO:N being a superior acceptor for charges generated independently within the P3HT. Instead our results indicate that nitrogen doping of the ZnO improves the efficiency of exciton dissociation at the heterointerface, resulting in the generation of a greater quantity of charge.

While the precise mechanism of the efficiency improvement warrants further investigation, it is noted that this enhanced dissociation occurs on an ultrafast timescale, with the difference between the SE/PIA signals for the ZnO and ZnO:N samples appearing within the first picosecond. This ultrafast charge generaton is consistent with that reported previously for P3HT on silicon, where the polaron absorption signal reached a maximum within 300 fs, which was attributed to highly delocalized excitations and ultrafast charge transfer from P3HT to silicon.[41]

The improvements in exciton dissociation and charge collection observed here with nitrogen doping are similar to those observed previously with the incorporation of an interfacial modifier (typically a self-assembled layer of an organic molecule) between the oxide and polymer.[12,42–45] These interfacial modifiers are likely achieving a similar effect as our enhanced photo-induced de-trapping of surface-localized species. The interfacial layers are known to passivate inorganic surface states by chemically interacting with surface dangling bonds,[46,47] such that the trapping of electrons and adsorption of gaseous species should be reduced. The modifiers are also often characterized by large electron affinities, which would further favor the removal of trapped electrons from the surface of the oxide, and they are known to be capable of inducing large changes to the work function on the oxide surface.[42,44,48] While our results have shown that ZnO can efficiently dissociate excitons when its properties are appropriately designed to expunge trapped electrons from its surface upon illumination, additional incorporation of interfacial modifiers also provides advantages, including influencing the polymer morphology on the oxide surface[12,28,49] and imparting a beneficial dipole that can reduce back recombination across the interface (e.g., recombination occurring via the formation of bound-charge-pairs).[42–44,50] Thus we suggest that the development of modifiers should focus on these key functions (improving polymer morphology and reducing recombination) in order to further enhance the performance of oxide/conjugated polymer donor-acceptor interfaces.

On the basis of our observations, we also fabricated similar devices in a manner that limited the presence of adsorbants on the surface of the oxide (and associated trapping of electrons). Devices were synthesized after exposing the oxide films to UV light for several minutes in a nitrogen glovebox prior to spin coating. Other devices were stored in nitrogen for several weeks prior to measurement in an attempt to remove any oxygen. Both strategies were found to result in ohmic devices with little photoresponse. This is consistent with previous studies where prolonged exposure of ZnO[51] or TiO_2_[19] to vacuum or inert gas was found to reduce device performance, which was attributed to the formation of detrimental oxygen vacancies and a correspondingly high electron density at the surface of the oxide, which enhances recombination.

Returning to the contrasting reports in literature, we note that previous instances where ZnO proved to be a good quencher of excitons in P3HT typically involved the chemical synthesis of ZnO within the P3HT phase.[3,6] This likely resulted in intimate contact and chemical bonding between the P3HT and ZnO phases, such that the trapping of surface species on the ZnO was limited. Studies that reported ZnO to be a poor exciton quencher typically involved “building-block” approaches, where the P3HT was deposited on top of a pre-fabricated ZnO surface prone to the trapping of surface species. These building-block approaches have a number of possible advantages, but our results clearly demonstrate that an oxide's properties need to be carefully tuned for use in such structures.

## 3. Conclusion

In light of contrasting reports, the ability of ZnO to act as a dissociating surface for P3HT was studied in this work. Our results indicate that the trapping of electrons on the surface of the ZnO inhibits its ability to dissociate excitons. However, we have shown that with proper design of the zinc oxide's properties, exciton dissociation at the oxide/P3HT interface can be effectively “turned on”. Doping the ZnO with a small amount of nitrogen to reduce its electron concentration results in dramatically improved surface properties. More efficient light-induced de-trapping of electrons is observed for the ZnO:N surface, which enhances exciton dissocation and electron transfer from the P3HT to the oxide. This model study provides useful insight into the process of exciton dissociation at oxide/conjugated polymer donor-acceptor interfaces. Furthermore, the ability to improve an oxide's surface properties through the incorporation of a bulk dopant has general applicability to the design of oxides for a variety of applications, including photovoltaics, photocatalysts, and charge injecting electrodes in hybrid polymer light-emitting-diodes.

## 4. Experimental Section

*Synthesis of Zinc Oxide*: Zinc oxide films were synthesized by AALD in a manner identical to that reported previously.[25] Diethylzinc was used as the zinc source and water as the oxygen source. Nitrogen was introduced by replacing the water precursor with an aqueous ammonia solution. Approximately 30% NH_3_ in water (puriss., 30–33% NH_3_ in H_2_O, Sigma-Aldrich) was used unless specified otherwise. The zinc and water precursors were bubbled using nitrogen flows of approximately 15 mL min^−1^ and 40 mL min^−1^ respectively, and the resulting vapors were delivered to flow channels adjacent to the substrate using a nitrogen flow of approximately 250 mL min^−1^. Inert nitrogen flow channels were located between the precursor flow channels to shield the adjacent precursor flows. The substrates were heated to 150 °C and passed underneath the precursor flows at a distance of approximately 60 μm and speed of 50 mm s^−1^. Approximately 500 zinc-water cycles were employed to deposit the films. Film thicknesses were measured using a Dektak Profilometer. All zinc oxide samples were annealed for 1 hour on a hot plate set to 350 °C, unless specified otherwise.

*Device Fabrication*: ZnO and ZnO:N films were deposited on commercial ITO/glass substrates (Colorado Concept Coatings) by AALD. Sepiolid P200 P3HT (Rieke Metals, Inc.) was dissolved in anhydrous o-xylene (97%, Sigma Aldrich) with a concentration of 15 mg mL^−1^, stirred overnight, and then spin-cast (600 rpm for for 45 s followed by 2000 rpm for 2 s) in a nitrogen glovebox. This was found to result in P3HT films approximately 100 nm thick. The deposited P3HT films were annealed for 15 min at 150 °C in the glovebox then contacts (approximately 5 nm of MoO_3_ (Testbourne Ltd.) and 50 nm of Ag) were deposited by thermal evaporation. For comparison, we also employed an alternative device synthesis recipe: approximately 300 nm thick P3HT films were deposited onto the ZnO and ZnO:N films by dissolving 4002-E P3HT (Rieke Metals, Inc.) in chlorobenzene (Sigma Aldrich) with a concentration of 30 mg mL^−1^, stirring in air overnight in the dark, then spin-casting in air at 600 rpm for 6 s, followed by 2000 rpm for 60 s. Prior to depositing the P3HT, the oxide films were sonicated in ethanol for 15 min, blown dry with compressed air, then heated at 120 °C for 10 min on a hotplate. After deposition of the P3HT, the samples were annealed at 150 °C for 15 min in air in the dark, and then similar contacts were thermally evaporated.

*Photovoltaic Measurements*: Current density–voltage measurements were taken using a Keithley 2636A source-measure unit under an Oriel 92250A solar simulator, at an intensity equivalent to 100 mW cm^−2^ after correcting for spectral mismatch. The device performance (particularly that of devices incorporating a ZnO:N/P3HT interface) was found to improve upon illumination and stabilize after 1–2 min. The *J*–*V* measurements presented here were obtained after approximately 3 min of illumination. We measured the photoresponse of the solar cells as a function of photon energy using light from an Oriel Cornerstone 260 monochromator. External quantum efficiencies were calculated from this, by comparing the solar cell photoresponse to the response from a reference diode.

*Time-Resolved Microscopic Surface Photovoltage Measurements*: Kelvin probe force microscopy was performed using an Agilent 5500 scanning probe microscope in amplitude modulation (AM) mode. Both the topography and surface potential were measured simultaneously, and feedback loops similar to those reported by Li et al. were employed.[52] We grounded the zinc oxide films by attaching a steel wire onto their top surface with silver paste and connecting the other end of the wire to the sample plate and grounding cable. A Pt-coated silicon probe with a resonant frequency of approximately 70 kHz and spring constant of approximately 2 N m^−1^ (Asylum Research, model AC240TM) was employed for the KPFM measurements. The sample was positioned on the same plate beneath the probe and a step-index multimode fiber optic cable (M35L02, Thorlabs) was fixed to the instrument and aimed at the sample at a distance of approximately 3 cm. The fiber was kept in an identical location for all experiments to ensure the same illumination intensity for all samples. A 4.1 mW M365FI fiber-coupled UV diode centered at 365 nm (Thorlabs) or 7.0 mW MWWHF1 fiber-coupled white-light diode (Thorlabs) was connected to the fiber optic cable to illuminate the samples. The instrument's laser was aligned with the probe's cantilever at the location of the tip and the probe brought down to the sample surface at 1 μm s^−1^. After scanning to tune the feedback loop controller and lock-in amplifiers in the instrument for measuring in KPM AM mode, all lights in the instrument were turned off (including the laser light) and the instrument casing closed so that the sample was left in the dark for 3 h. Subsequently, only the laser light was turned on to enable scanning. The UV or white light source was then turned on while scanning to measure the photo-induced change in the surface potential (surface photovoltage).

*Absorbance and Photothermal Deflection Spectroscopy Measurements*: Absorbance measurements of P3HT/ZnO and P3HT/ZnO:N bilayers, as well as neat P3HT films, were performed on Spectrosil B quartz substrates (UQG Ltd.) using a Hewlett-Packard 8453 UV−vis spectrometer. P3HT (Sepiolid P200) films approximately 10 nm and 50 nm thick were produced by spinning at 1000 rpm (1.5 mg mL^−1^ in o-xylene) and 2000 rpm (10 mg mL^−1^ in o-xylene) respectively. To measure the sub-band-gap absorption of the ZnO and ZnO:N, a custom-built photothermal deflection spectroscopy system was used. Photothermal deflection spectroscopy is a highly sensitive technique capable of measuring absorption 5–6 orders of magnitude weaker than the band edge absorption. Detailed description of the setup can be found in Kronemeijer et al.[53]

*Photoluminescence Measurements*: For photoluminescence measurements, zinc oxide films were deposited onto Spectrosil B quartz substrates and P3HT films approximately 10 nm thick were spin-cast in a nitrogen glove box from a 1 mg mL^−1^ solution of Sepiolid P200 P3HT in anhydrous o-xylene (1000 rpm for 45 s). The samples were fixed in an evacuated sample holder and exposed to simulated solar illumination for 3 min immediately prior to measurement of the photoluminescence. The excitation source was a PicoQuant LDH400 pulsed 10 MHz diode laser (*λ*_excitation_ = 470 nm, 80 ps full width at half maximum) and the photoluminescence spectra were measured using a 500 mm spectrograph (SpectraPro2500i, Princeton Instruments) combined with a CCD camera (Acton 100-F, Princeton Instruments).

*Transient Absorption Measurements*: For transient absorption measurements, zinc oxide films were deposited onto Spectrosil B quartz substrates and P3HT films approximately 50 nm thick were spin-cast. Sub-picosecond transient absorption measurements were carried out using approximately 100 fs pulses at 530 nm as the excitation source and broadband pulses between 500 and 800 nm as the probe. Both pulses were generated from the output of an 800 nm, 3.5 W, 1 kHz Solstice amplifier system (Newport) with a TOPAS (Light Conversion) and home-built non-collinearly pumped optical parametric amplifier respectively. A more detailed description of the setup was reported by Moor et al.[54] A dual line camera (Stresing Entwicklungsbüro) was used as the detection system. The excitation wavelength was set to a low 1.5 μJ cm^−1^ fluence as to minimize multi-exciton-decay processes.

## Supporting Information

As a service to our authors and readers, this journal provides supporting information supplied by the authors. Such materials are peer reviewed and may be re-organized for online delivery, but are not copy-edited or typeset. Technical support issues arising from supporting information (other than missing files) should be addressed to the authors.
