# Supplementary material for: Improved Exciton Dissociation at Semiconducting Polymer:ZnO Donor:Acceptor Interfaces via Nitrogen Doping of ZnO
Source: Adv Funct Mater. 2014 Mar 7;24(23):3562–70. doi: 10.1002/adfm.201303994 (PMC4228972; doi:10.1002/adfm.201303994)
Supplement: Supplementary file 1 — Supplementary [file adfm0024-3562-SD1.pdf]

Copyright WILEY-VCH Verlag GmbH & Co. KGaA, 69469 Weinheim, Germany, 2014.

ADVANCED FUNCTIONAL MATERIALS

Supporting Information

for *Adv. Funct. Mater.,* DOI: 10.1002/adfm.201303994

Improved Exciton Dissociation at Semiconducting Polymer:ZnO Donor:Acceptor Interfaces via Nitrogen Doping of ZnO

Kevin P. Musselman, Sebastian Albert-Seifried, Robert L. Z. Hoye, Aditya Sadhanala, David Muñoz-Rojas, Judith L. MacManus-Driscoll, and Richard H. Friend*

Copyright WILEY-VCH Verlag GmbH & Co. KGaA, 69469 Weinheim, Germany, 2013.

Supporting Information

Improved exciton dissociation at semiconducting polymer:ZnO donor:acceptor interfaces *via* nitrogen doping of ZnO

Kevin P. Musselman, Sebastian Albert-Seifried, Robert L. Z. Hoye, Aditya Sadhanala, David Muñoz-Rojas, Judith L. MacManus-Driscoll, and Richard H. Friend*

<u>Section S1 – Estimation of the occupancy of states in the zinc oxide conduction band</u>

The density of states near the conduction band edge of a semiconductor ρ_c can be approximated as a function of energy E as:

$$\rho_c(E) = \frac{(2m_c)^{3/2}}{2\pi^2\hbar^3}(E - E_c)^{1/2} \quad (1)$$

where m_c, $\hbar$, and E_c are the effective mass of electrons in the conduction band, Plank's constant, and the energy at the conduction band edge respectively[1]. An effective mass of $0.28\,m_e$ was used for ZnO, where m_e is the electron mass[2]. The black line in Figure S1 shows the approximate density of states in the ZnO conduction band.

The occupancy of the conduction band states can be approximated by the Fermi function:

$$f(E) = \frac{1}{\exp\left[\frac{E - E_f}{kT}\right] + 1} \quad (2)$$

where E_f, k, and T are the Fermi energy, Boltzmann constant, and temperature respectively[1]. Combining equations (1) and (2), the E_f corresponding to various carrier concentrations can be estimated. This E_f can then be inserted into equation (2) to model the occupancy of conduction band states for different ZnO carrier concentrations, as shown by the shaded regions in Figure S1.

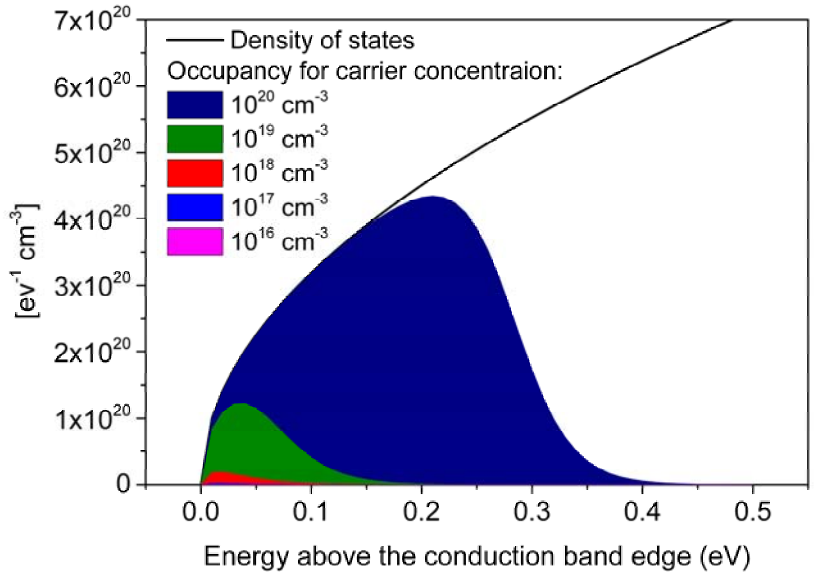

Figure S1: Estimated density of states in the conduction band, and occupancy of these states for different ZnO carrier concentrations.

[1] B.E.A. Saleh and M.C. Teich, *Fundamentals of Photonics*, John Wiley & Sons, Inc., 1991.

[2] D.C. Reynolds, D.C. Look, B. Jogai, *Solid State Commun.* 99 (1996) 873-875.

Section S2 - UV-vis absorbance measurements of P3HT on ZnO and ZnO:N

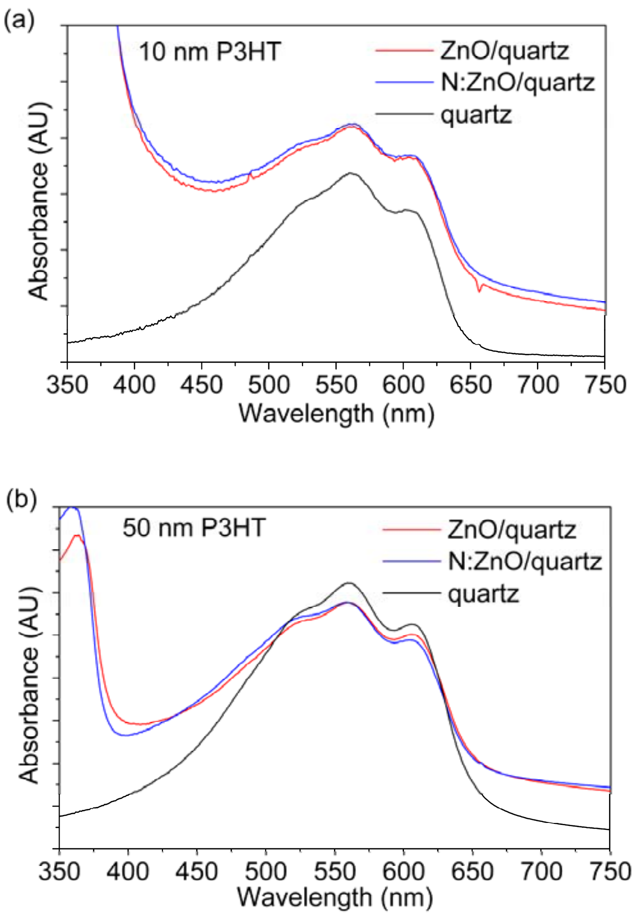

Figure S2: Absorbance measurements of P3HT films approximately (a) 10 nm and (b) 50 nm thick on quartz substrates with ZnO, ZnO:N, and no surface coatings. No difference in the absorption spectra is observed for P3HT on the ZnO and ZnO:N surfaces. Similarly, little difference in the absorbance spectra is observed for P3HT on the oxide surfaces versus the neat quartz glass, which may be due in part to the smooth surface of the films deposited by AALD.

Section S3 - Photovoltaic characterization of devices produced using alternative synthesis conditions

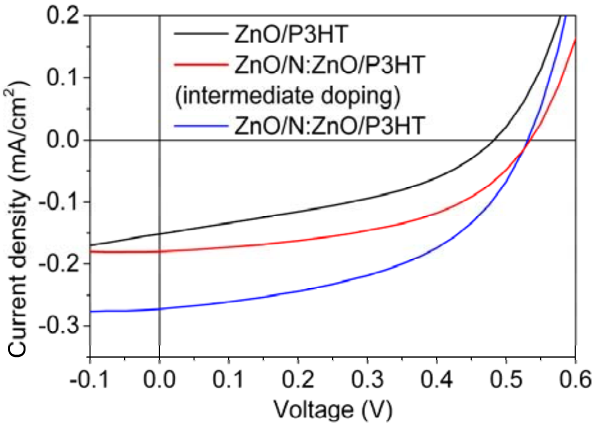

Figure S3: Current density-voltage measurements under simulated solar illumination for ZnO/P3HT and ZnO/ZnO:N/P3HT devices fabricated using the alternative recipe detailed in Section 2.2. The thickness of the oxides films was approximately 65 nm ZnO and 20 nm ZnO:N, similar to that used in the devices of Figure 2c-d. The ZnO:N coating with an intermediate doping was produced by using a 10% NH_3 precursor, rather than 30%.

Section S4 – Surface photovoltage measurements

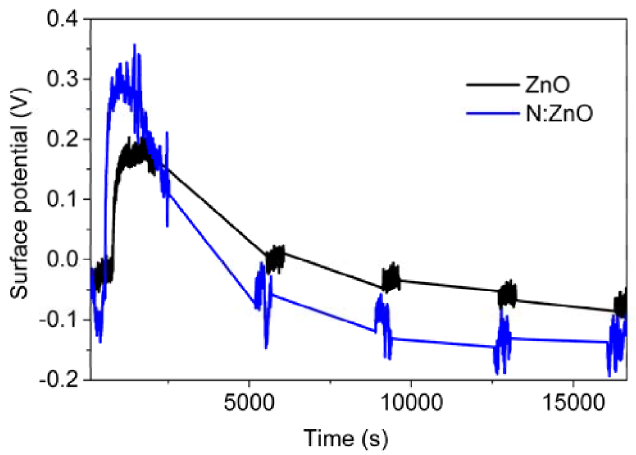

Figure S4a: Decay of surface potential (relative to Pt KPFM tip) for ZnO and ZnO:N samples after turning off the UV illumination.

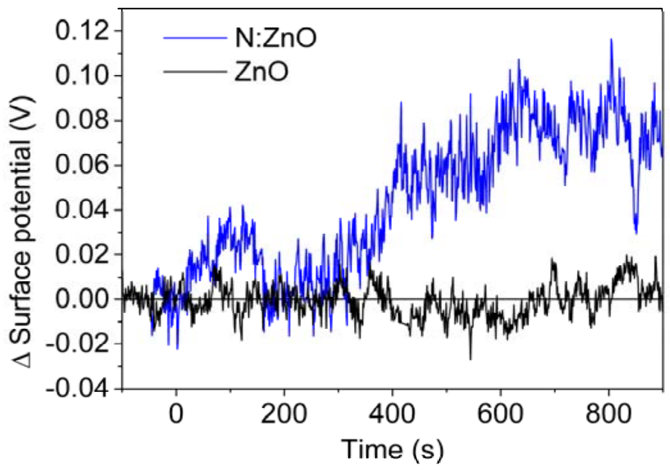

Figure S4b: Surface potential measured for ZnO and ZnO:N surfaces when illuminated with white light.

Section S5 – Photothermal deflection spectroscopy measurements of ZnO and ZnO:N

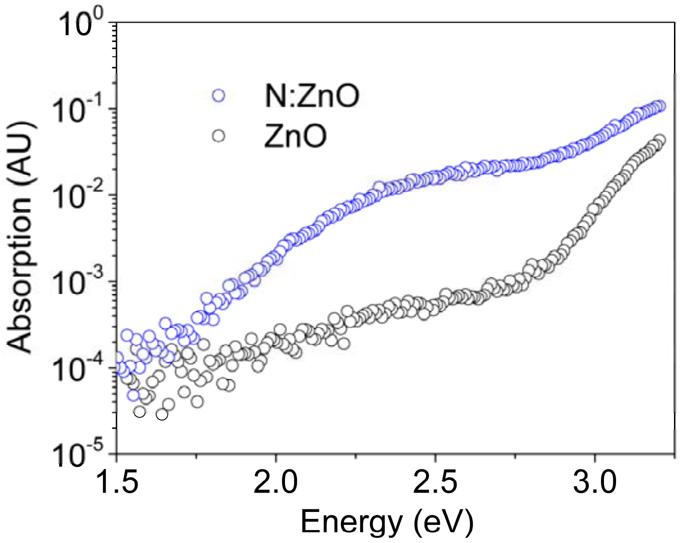

Figure S5: Absorption of ZnO and ZnO:N films, as measured by photothermal deflection spectroscopy.

Section S6 – Transient absorption spectroscopy measurements

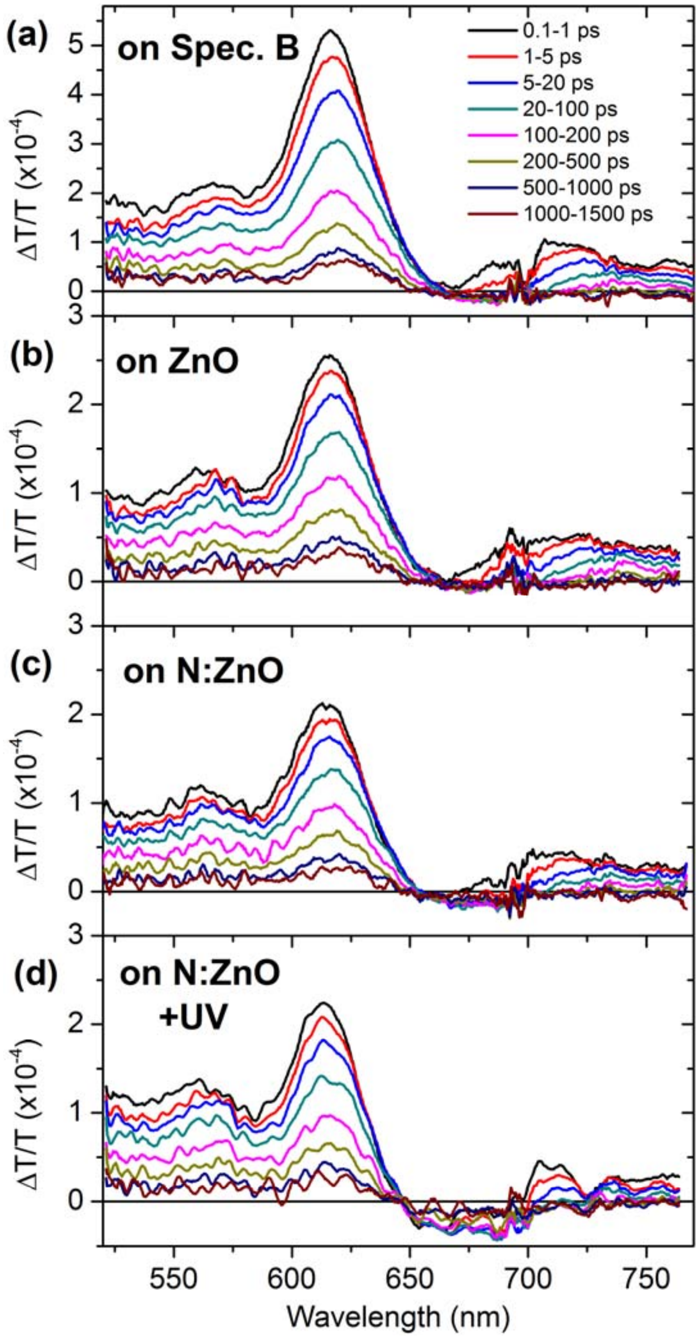

Figure S6: Transient absorption spectra for approximately 50 nm thick P3HT films on (a) Spectrosil B (quartz glass), (b) ZnO, (c) ZnO:N, and (d) ZnO:N after exposure to UV illumination for 3 minutes.
